# Supplementary material for: Hyaluronic acid on the urokinase sustained release with a hydrogel system composed of poloxamer 407: HA/P407 hydrogel system for drug delivery
Source: PLoS One. 2020 Mar 11;15(3):e0227784. doi: 10.1371/journal.pone.0227784 (PMC7065803; doi:10.1371/journal.pone.0227784)
Supplement: S1 Fig — (DOCX) [file pone.0227784.s001.docx]

Figure S1.

(A)


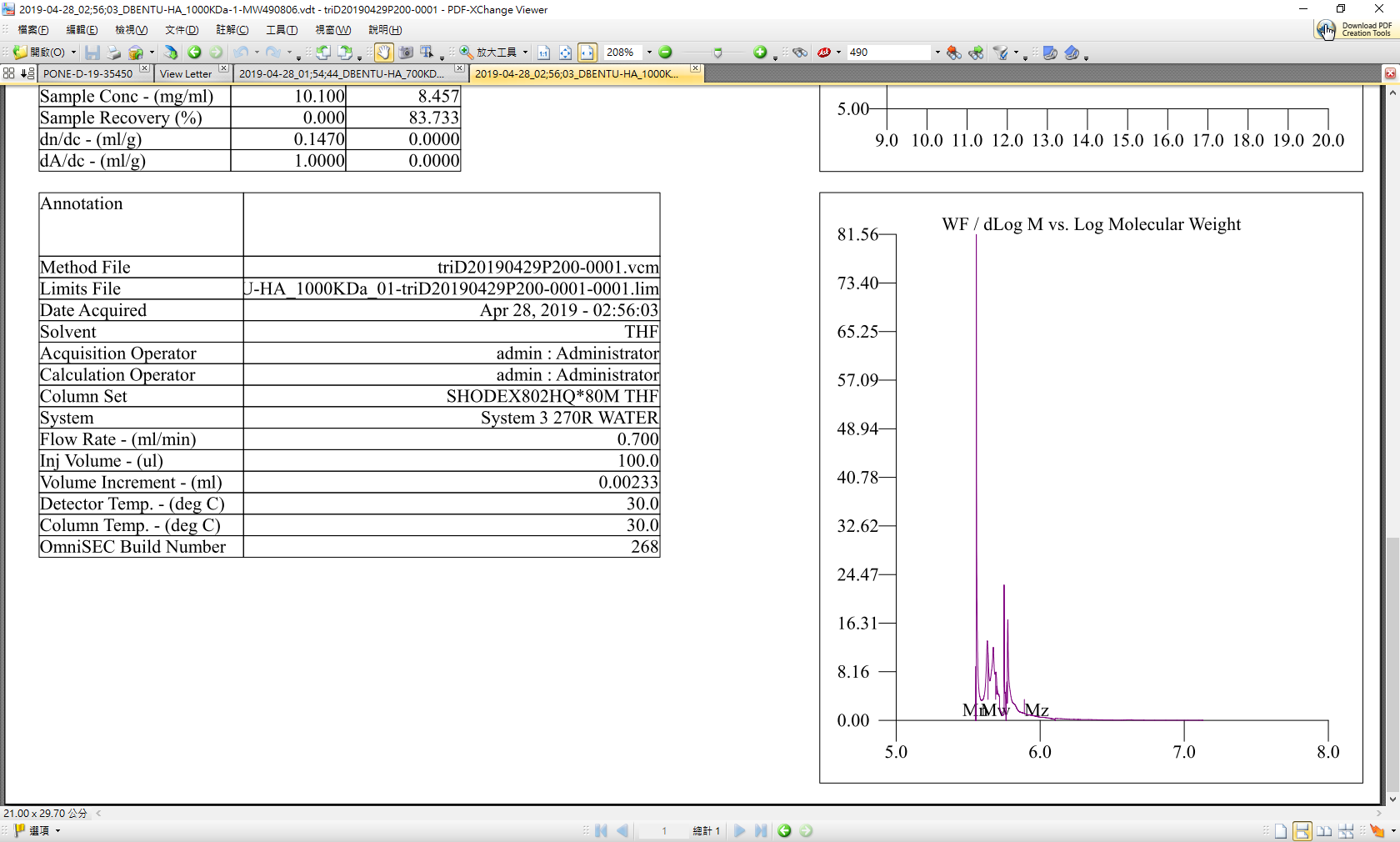

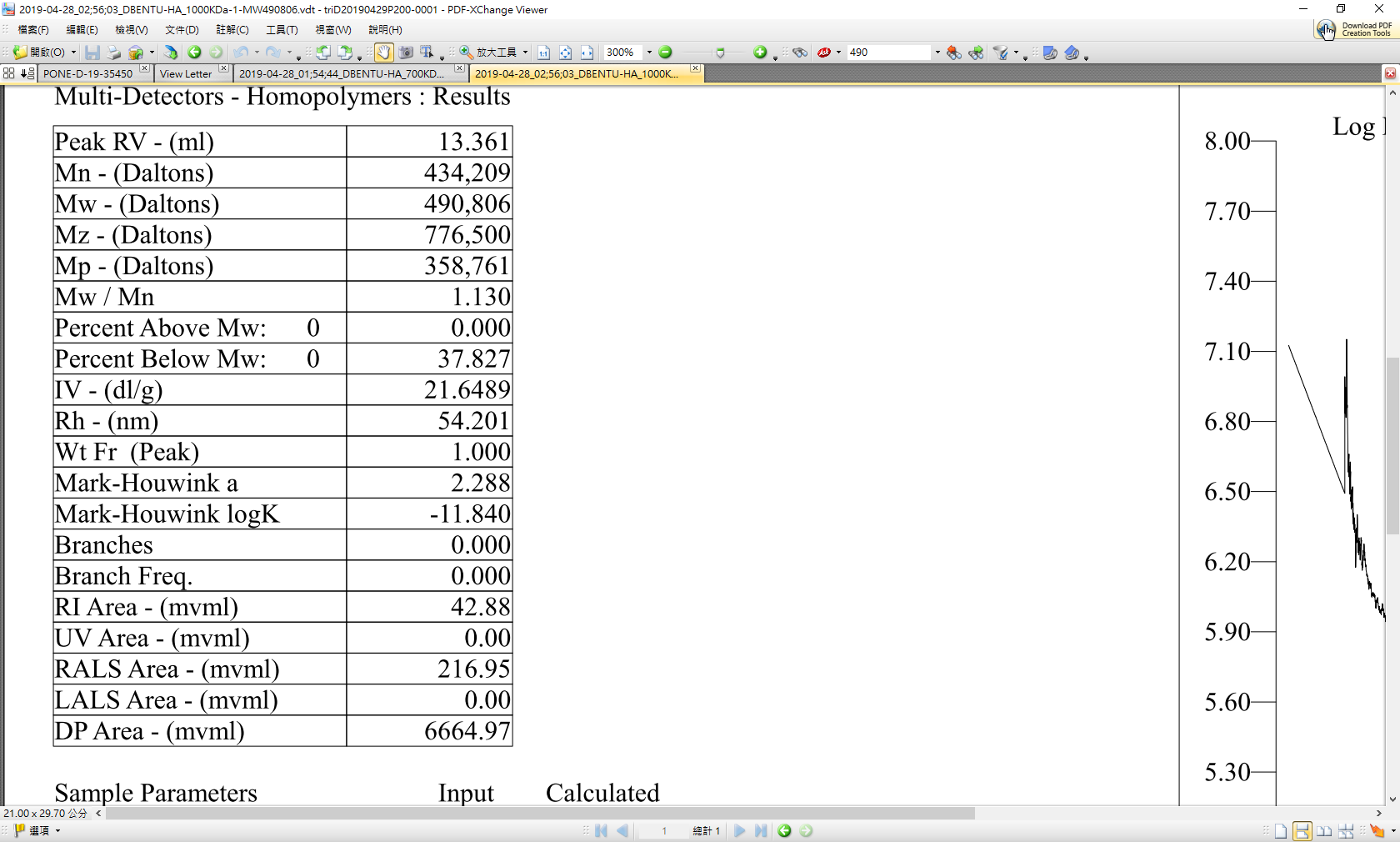


(B)


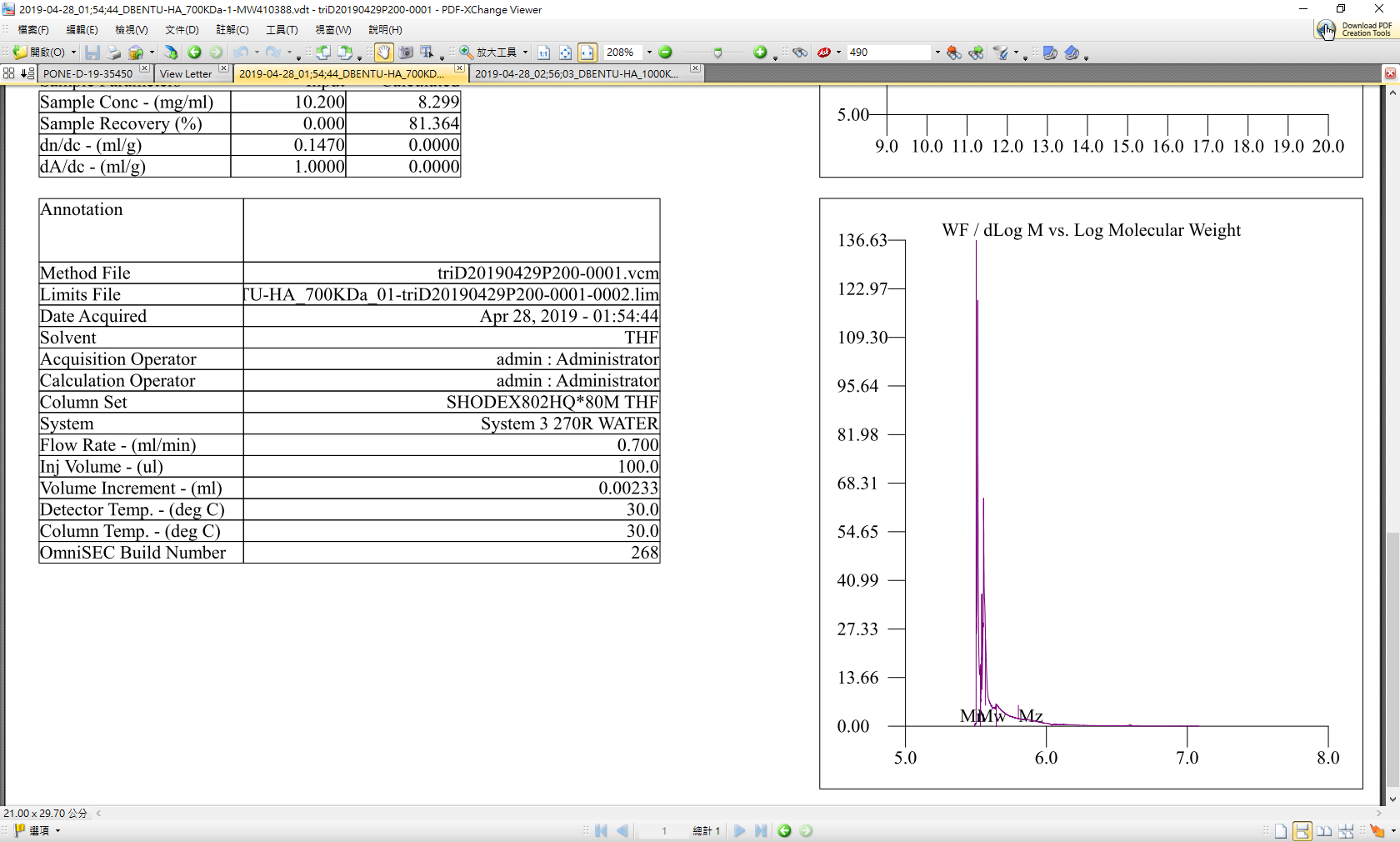

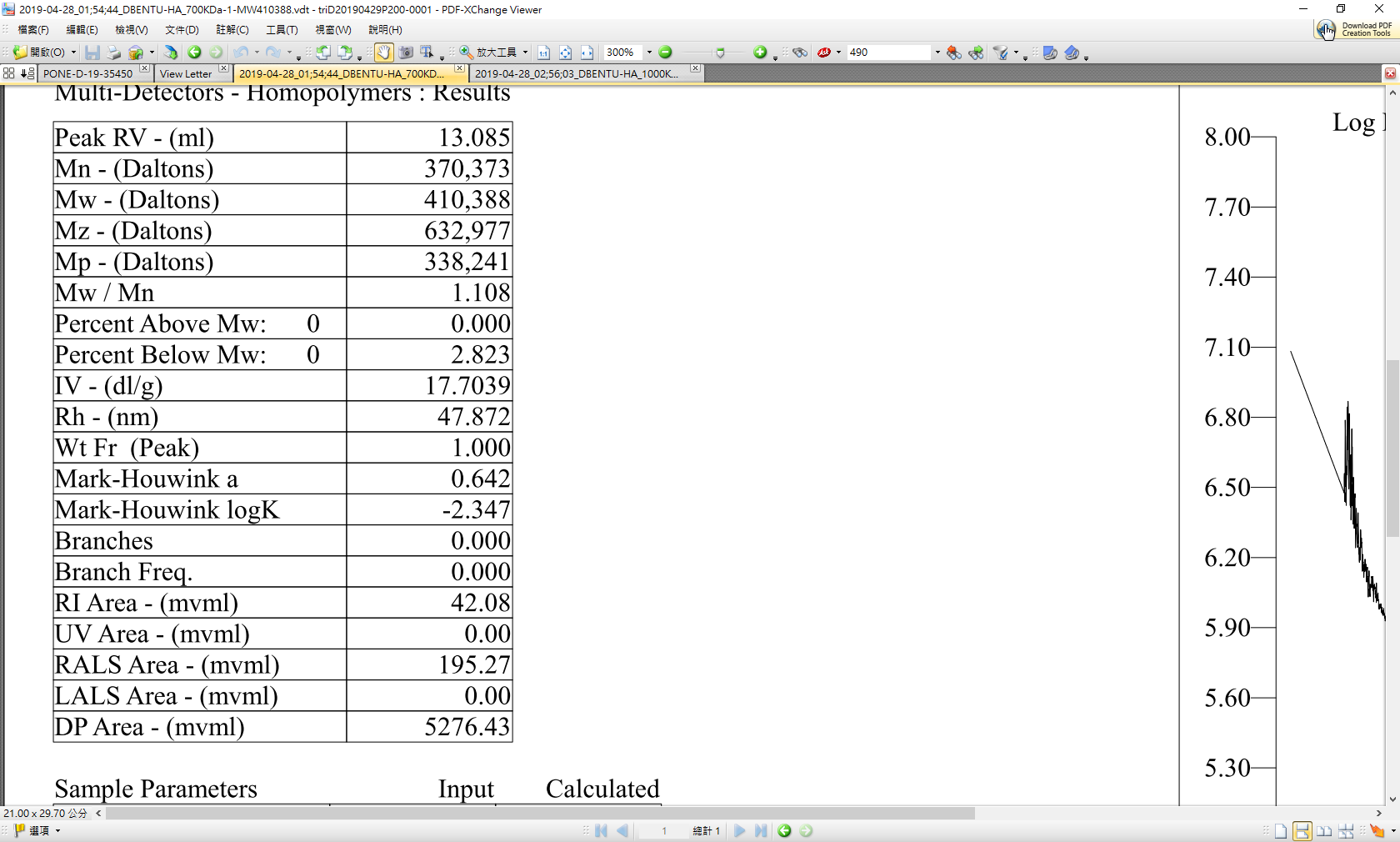


Figure S1. The Gel Permeation Chromatography(GPC) chromatogram

of (A)490kDa and (B)410kDa Hyaluronic acid(HA).
