# Supplementary material for: Hyaluronic acid on the urokinase sustained release with a hydrogel system composed of poloxamer 407: HA/P407 hydrogel system for drug delivery
Source: PLoS One. 2020 Mar 11;15(3):e0227784. doi: 10.1371/journal.pone.0227784 (PMC7065803; doi:10.1371/journal.pone.0227784)
Supplement: S3 Fig — The gel dissolution time decreased markedly in urokinase-loaded 23% P407 in the comparison with the non-urokinase-loaded gels. (DOCX) [file pone.0227784.s003.docx]

Figure S3.

(A)
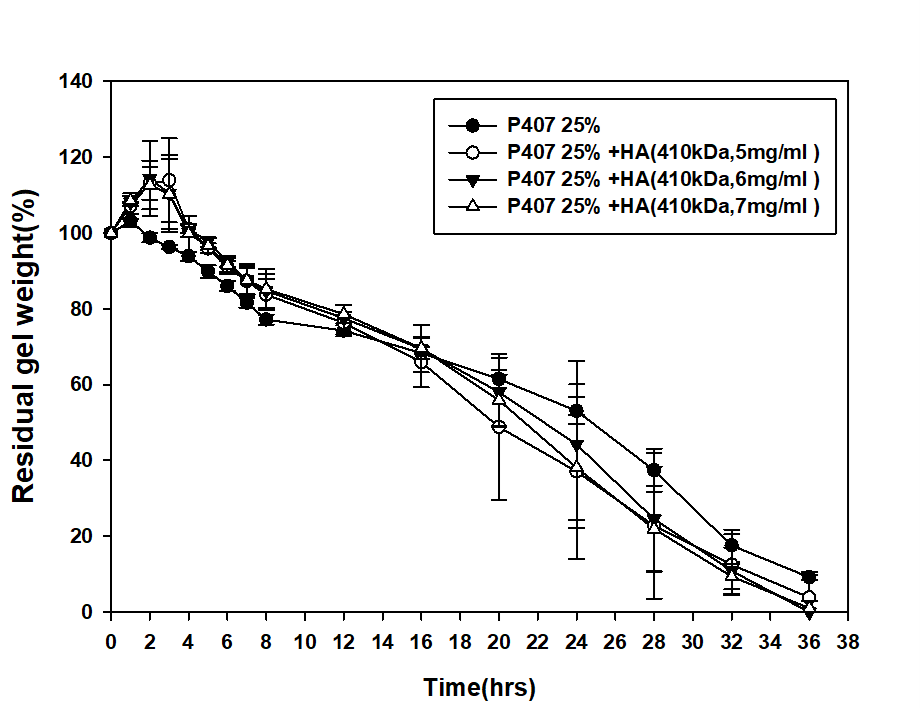


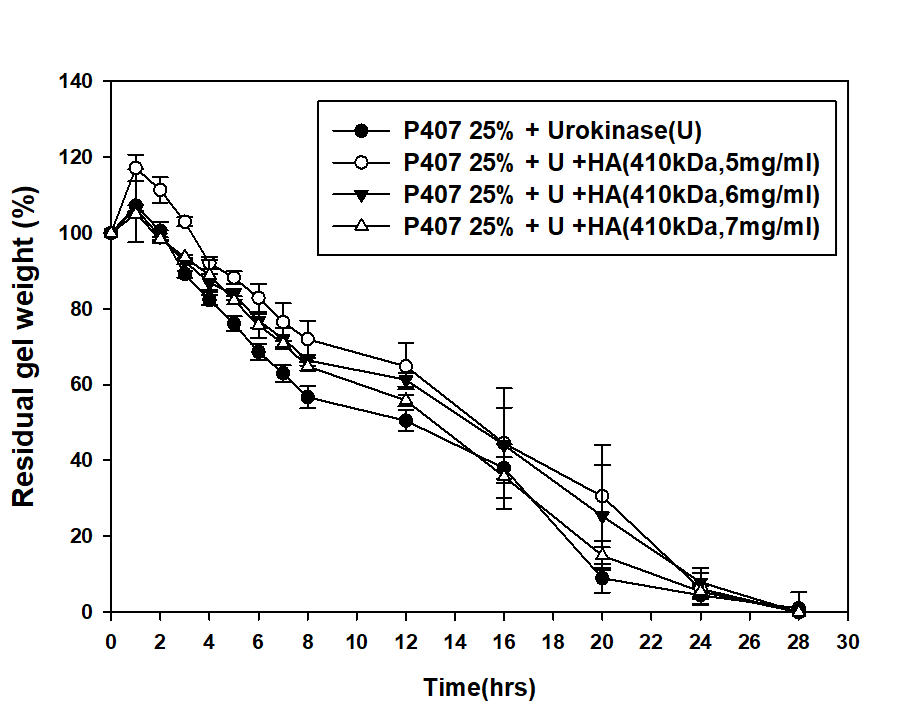
(B)

Figure S3. Gel dissolution profilesof(a) 25% and (b) 25% urokinase-loaded P407 with HA. The gel dissolution time decreased markedly in urokinase-loaded 23% P407 in the comparison with the non-urokinase-loaded gels.
