# Supplementary figures and images for: Hyaluronic acid on the urokinase sustained release with a hydrogel system composed of poloxamer 407: HA/P407 hydrogel system for drug delivery
Source: PLoS One. 2020 Mar 11;15(3):e0227784. doi: 10.1371/journal.pone.0227784 (PMC7065803; doi:10.1371/journal.pone.0227784)

Figure S4.


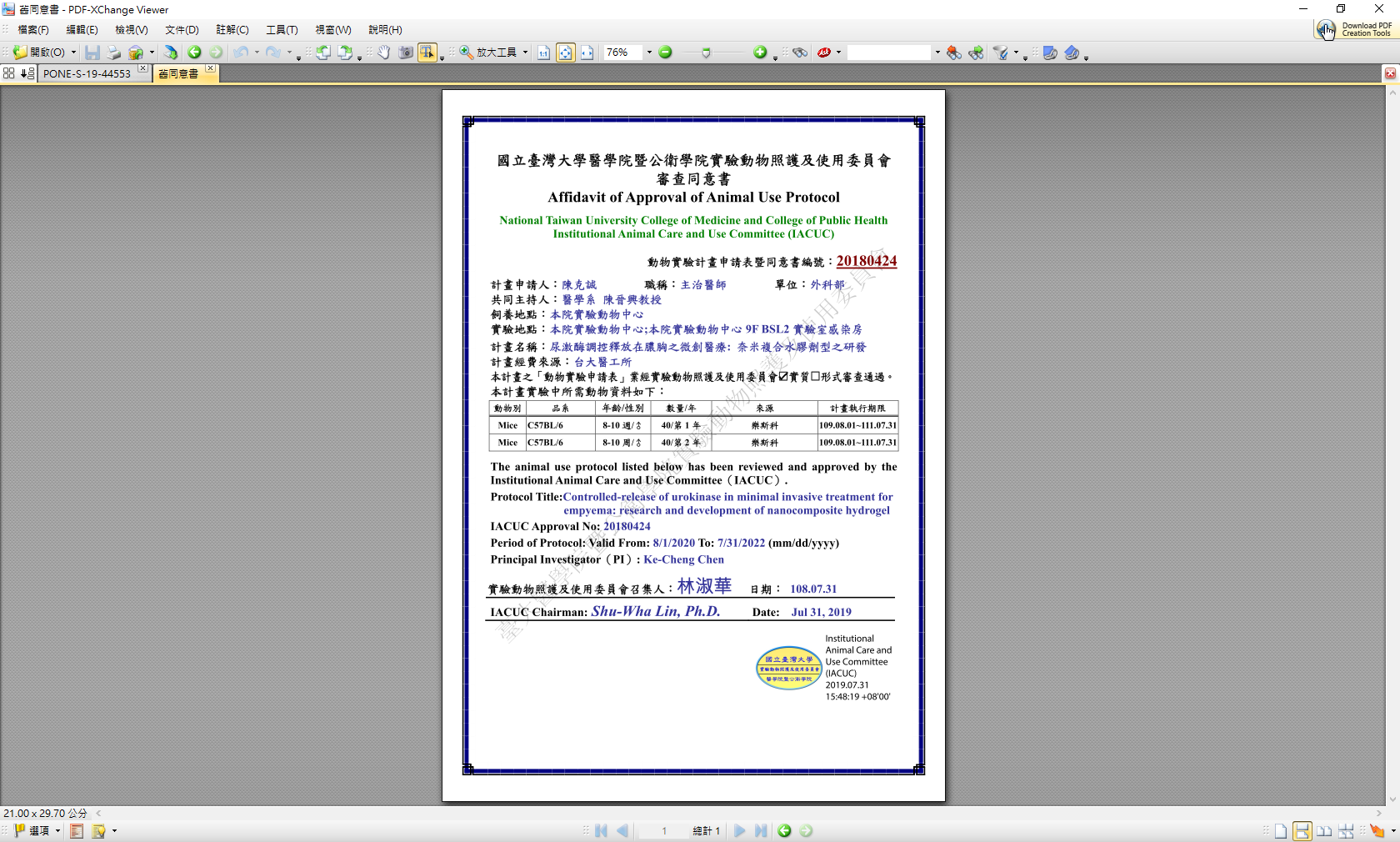


Figure S4. Affidavit of Approval of Animal Use Protocol.

Supplement: S4 Fig — (DOCX) [file pone.0227784.s004.docx]
